# Searching for the New Behavioral Model in Energy Transition Age: Analyzing the Forward and Reverse Causal Relationships between Belief, Attitude, and Behavior in Nuclear Policy across Countries

**DOI:** 10.3390/ijerph19116772

**Published:** 2022-06-01

**Authors:** Byoung Joon Kim, Seoyong Kim, Youngcheoul Kang, Sohee Kim

**Affiliations:** 1 Department of Public Administration, Kookmin University, Seoul 02707, Korea; kimbj@kookmin.ac.kr; 2 Department of Public Administration, Ajou University, Suwon 16499, Korea; 3 Graduate School of Public Administration, Korea University, Sejong 30019, Korea; 4 Research Center for Energy Transformation Policy, Social Science Research Institute, Ajou University, Suwon 16499, Korea; shkim219@ajou.ac.kr

**Keywords:** nuclear energy perception, the effect of trust, conflict resolution model, comparative analysis

## Abstract

This study aims to analyze the forward/reverse causal relationships between belief (risk perception), attitude (judgment), and behavior (acceptance). A traditional view stresses forward causal relationships between the three variables. However, recently, several studies have reported the possibility of reverse causal relationships between them. Based on survey data collected from 1027 Korean/Japanese participants, here we test not only the forward or reverse relationships between these three variables, but also how such causal relationships depend on the trust and country contexts (Korea and Japan in this study). The results showed that, first, not only a general forward causal relationship but also reverse causal relationship exists between belief, attitude, and behavior. Second, there exist the moderated mediation and mediated moderation effect of trust in government and media across two countries. Third, the effects of trust in government and media work significantly overall. However, the patterns of interaction effects differ between two countries. The level of trust in the government influenced the belief and attitude of citizens in Japan more than in Korea. However, the level of trust in the media showed opposite results.

## 1. Introduction

Discussions on the future of nuclear power have been triggered across countries after the accident at Japan’s Fukushima nuclear power plant in 2011 [1]. Public opinion on nuclear power is a major determinant of whether it is used to generate energy [2]. After a major nuclear power plant accident, such as the Three Mile Island accident in the United States in 1979 and the Chernobyl accident in the Soviet Union in 1986, public attitudes toward nuclear energy had changed significantly [3]. Previous studies have noted that public’s perceived risk toward nuclear power has increased, and there have been greater levels of psychiatric illness with specific signs of depression, fear, and hostility about it [4,5]. Consequently, psychometric analysis concludes that nuclear power has been recognized as something with a frightening and uncertain danger in the minds of citizens [6,7].

Moreover, since the Fukushima accident in 2011, public polls in many nations have found that people worried about nuclear power. In the past, neighboring nations such as Korea and China backed conventional rational nuclear policymaking based on scientific foundations. However, after the Fukushima accident, as the risk perception of nuclear power plants has increased, logic of rational model in policymaking has begun to be questioned [8,9,10]. Rationality is one of the long-running debated issues in the social sciences. Generally, individuals choose their behaviors based on a desired outcome, according to the rational and instrumental models which are usually employed in policy decisions for nuclear energy and public health [11,12]. However, citizens’ opinions on high-risk issues such as nuclear power are complicated. As the nature of concerns about risky objects such as nuclear power varies according to people’s risk judgment, their views are flexible and unsettled. In this vein, many researchers have begun to pay the attention to the notion of “bounded rationality,” not complete objective conjunctions [9,11].

To beyond the rational model, it is time to find the new model to explain people’s way of thinking about nuclear issues. To obtain a more comprehensive understanding of attitudes toward nuclear energy and other risk issues, first, scholars should prioritize inferences aimed at identifying not only the sources of current attitudes, but also the causal models explaining the relationships between variables [12,13,14,15,16,17,18,19,20]. When judging nuclear power, people use the causal model in their minds. For example, belief and attitude precede behavioral intention, and intention to action precedes the behaviors [13]. As a result, it is necessary to examine relationships between variables that include belief (risk perception), attitude (judgment), and behavior (not-acceptance, more exactly actions against nuclear power in our analysis). Second, it is necessary to search for a causal model that can be universally applied beyond context. In particular, there is demand for a universal causal rule that can be applied across national contexts. To this end, it needs the comparative study by which the universality of cause rule can be verified. Third, in research about nuclear acceptance, the research should shift the focus from the simple causal effect to more elaborate one, e.g., the moderating or mediating effect, both of which are indirect rather than direct effect. In order to generalize and develop these indirect effects, cross-context comparison studies are required. However, there are few studies of this kind.

This study focuses on three research topics. First, we examine the more extended causal relationships. In order to more convincingly explain behaviors such as acceptance of specific energy, it is necessary to test the causal model composed of more steps beyond simple relationships between two variables, i.e., independent variable and dependent one. Following a psychological or behavioral economic analysis, we examined the forward and the reverse relationships between belief, attitude, and behavior. The field of behavioral economics insists that the level of citizens’ cognition, attitude, and behavior can be explained by bounded rational approaches [21]. Second, this study conducted a comparative study between Korean and Japanese to explore the universality of the causal model. This study’s data comes from national surveys on citizens’ perceptions of nuclear energy conducted in 2018 in both Korea and Japan. Using the integrated model by Muller et al. [22], this study found three different models of interaction between three components such as belief–attitude–behavior: (1) high involvement model, (2) low involvement model, and (3) hedonic model. Third, this study verifies an indirect effect such as the mediated moderation and moderated mediation effects by focusing on trust in government or media.

This paper is divided into four sections. The first section examines series of previous studies on belief (perceived risk), attitude (judgment), and behavior (acceptance). Next, this study provides an overview of the research method used to apply the Integrated Model by Muller et al. [22]. Third, we provide the analysis results. We will articulate how analysis results can become a bridge spanning the relation of three different models. Finally, this study concludes by offering research implications and its limitations.

## 2. Theoretical Background

### 2.1. Belief: Perceived Risk on Nuclear Power

Belief is a kind of fundamental orientation which people hold. It is expressed as emotional response such as the fear and perceived risk. We focused the perceived risk as a belief. Perceived risk about nuclear power has changed as the time and context vary. In response to the energy crisis of the 1970s, various countries initially began building nuclear power plants that have contributed to the stability of electricity [23]. Concerning the long-term stability of the nuclear industry, the consensus seems to point to a business-as-usual scenario. However, the consensus that nuclear power is a significant part of the solution to energy problems has faded away fast after Japan’s Fukushima nuclear accidents. Recent anti-nuclear protests occur in different countries, which demonstrate global apprehension about nuclear power, offer important lessons about how we consider citizens’ subjective reactions to nuclear energy.

Generally, the public has a perceived risk about nuclear energy. This perceived risk has been regarded as a crucial independent variable for determining the level of acceptance and policy satisfaction [24,25]. The Fukushima nuclear accident in Japan has made the acceptance of nuclear power precarious. Many of the proposed new reactors have been postponed while the situation in Japan has been analyzed. The orientation of new nuclear power plants seems to be increasingly cautious; even if they are generally non-threatening, the public remains cautious about the potential risk from them [26]. The public’s risk perception of nuclear energy is an important factor in determining national energy policy [27]. 

Risk is relatively constructed concept; countries’ different environments can affect people’s risk perception. As behaviors and perceptions are strongly influenced by historical and cultural structures, each country’s specific context affects the perceived risks of nuclear energy. Individuals from different cultures have diverse risk perceptions, not only because of individuals’ perceptual differences, but also because of objectively different risk environments [28]. Therefore, since the contextual differences may influence the variance in subjective risk perception, citizens across diverse countries will have different perspectives on the risk of nuclear energy.

Empirical studies on risk perception have indicated that a number of citizens have a much more different view of risk based on aspects such as the hazard’s familiarity, whether exposure to the hazard is discretionary, the magnitude of accidents it could possibly cause, disparities between risks and benefits, and the long-term consequences [29]. Thus, in order to derive a more generalized law beyond individual differences, it needs a comparative analysis to show the perceived risk of nuclear power. Nuclear risks, for example, are well identified in Japan but are comparatively unknown in the United States [30].

In Japan, according to the study by Kanda et al. [31] on the perceived risk of nuclear power and other risks, Japanese people’s risk perceptions have been consistent over the last 25 years (1983–2007), regardless of gender, age, and occupation. For example, female clerical staff have consistently judged nuclear power as the highest risk during the last 25 years, while researchers’ understanding has fluctuated with events such as the Chernobyl accident. This means that citizens learn to accommodate scientific technologies with low risks in exchange for high benefits, except in the case of nuclear power. Since nuclear power raises risk, at the same time, actions against it have benefits. Thus, nuclear power was regarded as high-risk by the Japanese even before the Fukushima nuclear power plant accident in March 2011. In addition, previous studies have focused on determining how perceived risk affects satisfaction or acceptance of nuclear power [11,24].

On the other hand, in Korea, citizens have traditionally shown mixed belief when there are positive or negative emotion toward nuclear energy [25]. When positive benefits related to nuclear technology occur, such as the export of nuclear plants, the public expresses positive emotional responses. However, negative accidents involving nuclear energy, such as the Fukushima disaster, have a profound impact on popular emotions. Because the perceived risks of nuclear energy are predicted to be irreversible and dangerous to the public, this negative emotion is likely to persist.

While Japan and South Korea (Korea, hereafter) are neighboring countries in Northeast Asia, their cultural, political, social, and economic characteristics are considerably different [31]. Due to these contextual differences, the perceived risk of nuclear energy is varied. Such difference brings out the various causal relationships among belief (perception), attitude (judgment), and behavior.

### 2.2. Attitude: Judgement toward Nuclear Energy Policy

Attitude as a proximal byproduct of judgment is a kind of evaluation. Satisfaction or positive/negative response toward specific objects are regarded as one attitudinal evaluation dimensions. 

Attitude appears not only at individual but also at collective level. Nuclear energy can be defined as low-likability, high-cost objects that affect not only the environment, but also society, with effects beyond the individual [32]. Nuclear energy sources have been discussed and public negative response is promoted because of both the possible environmental consequences and the existence of the risks involved. Such collective attitudes, however, are strongly dependent on limited expertise, the experience of policy communication, and media coverage. Knowledge is key component for judgment in decision making [33,34,35,36]. For example, since knowledge is one component of belief, it is presumed that knowledge influences attitudes indirectly through salient beliefs [34]. According to Kuklinski et al. [35], more objective experiences were linked to more positive attitudes toward nuclear energy. Experts and ordinary people have different views on radioactivity and nuclear energy facilities. For experts, nuclear energy is not a complex area to manage; the risks and chances of accidents are minimal and manageable. There are alternative solutions available that are secure and practically viable. However, ordinary people exhibit fear and apprehension toward nuclear energy, as well as a sense of risk surrounding the installations, which contributes to the prevalent resistance to radiation facilities (CF., NIMBY).

Additionally, conflicts come from different attitudes. There is little agreement over judgmental attitudes on risk objects such as nuclear energy. This is the most difficult thing to resolve the different attitudes in terms of conflict mediation without meaningful and effective contact. In this respect, previous studies confirmed that the most severe negative effects on attitude at Chernobyl were caused by the fear of radiation, not radiation itself. Even those that are geographically distant will have been impacted by this aspect. For example, the paper indicated that abortions increased by approximately 2500 in Greece because of an unfounded fear of radiation from Chernobyl [37]. Based on those findings, the individual’s judgmental attitude toward nuclear energy can be used to help predict their behavior; the more positive the attitude toward the behavior, the more likely it is that the individual intends to perform the behavior (accepting or protesting actions).

There are differences in attitude and its change toward nuclear energy issues between Korea and Japan. In Japan, the relationship between knowledge and risk perception was found to be moderate among Japanese respondents, even after radioactive gas release in the Tokai village power plant [38]. However, unlike the previous trends of attitude toward nuclear energy, the impact of the Fukushima accident was much greater than that of previous nuclear-related accidents such as Tokai. Kitada [39] analyzed surveys conducted over the last 30 years and reported that (1) negative attitudes toward nuclear power generation, which ranged between 20% and 30% over the previous 30 years, increased to 70% in the four to six months following the Fukushima incident; (2) many people opposed future replacements or new nuclear power plant construction; and (3) while considering options for energy production, many people opposed nuclear energy. According to 2015 survey conducted by the pro-nuclear Japan Atomic Energy Relations Organization, 47.9% of respondents said nuclear energy should be phased out gradually, while 14.8% said it should be phased out immediately. Only 10.1% believed nuclear technology should be continued, and only 1.7% believed that it should be expanded [40]. In particular, the challenge faced by the Japanese government is the legacy of poor communication, concealment, and misinformation about nuclear safety issues at the Fukushima plant. Therefore, trust has been damaged. To change the attitude, it is necessary to make significant efforts to regain public trust and encourage interactive communication on nuclear policy.

In Korea, previous energy policies suggested that nuclear energy would maintain a steady share of the country’s energy balance. According to a previous survey on nuclear energy, positive attitudes toward nuclear safety peaked at 60% in 2009, fell to 34% in 2012, and have yet to reach 40%. These numbers are unsurprising in light of the Fukushima accident [41]. The Fukushima accident has had a huge impact on attitude toward nuclear policy, but in Korea, the impact was considerably higher than in Japan. However, Korea’s stance toward nuclear power is significantly different from that of Japan. It does not advocate for an outright restriction on nuclear plants on a global level, and it has entered the global market for nuclear power plant builders [42]. This trend became particularly evident in 2011, when KEPCO, Korea’s nuclear research leader, signed an agreement with the United Arab Emirates to construct four nuclear reactors.

Therefore, even though the Fukushima accident has had a huge influence on the attitudes toward nuclear energy in Japan and Korea, people in both countries have shown different judgments on further steps in nuclear energy policy.

### 2.3. Behavior: Acceptance of Nuclear Energy Policy

Belief and attitude influence behavior, i.e., acceptance. Our analysis will focuse not-acceptance behavior, more exactly actions against nuclear power. As ‘acceptance’ is a term applied to a person’s recognition of information, it requires commitment, which indicates that behavior is maintained or modified in pursuit of personal beliefs and objectives [43]. Thus, articulated attitudes contribute to changing specific behaviors, especially in the event of an accident. As Zaller [44] suggested, “people tend to resist arguments that are inconsistent with their predispositions, but they do so only to the extent that they possess the contextual information necessary to perceive a relationship between the message and their predisposition.” Acceptance of a message is conceptualized as a function of the interplay between perception and predisposition [45], which provides the clues to change the behavior. Thus, acceptance may contribute to behavioral changes.

Acceptance is a kind of choice action, depending on the perception of risk in nuclear issues. In this respect, effective communication helps establish strong relationships between people’s risk perception and health behavior. Most research pertaining to risk relies on the assumption that the perception of risk correlates with behavior [46]. In addition, other research concentrates mainly on perception of risk as a predictor of behavioral intentions [47,48], supported by the evidence that intentions are positively correlated with actual behavior [49]. When the initial appraisal of a threat results in cognitive uncertainty, people become increasingly motivated to engage in information-seeking behavior [50].

From the point of view of acceptance, in Japan, citizens continue to oppose nuclear energy policies. In the last decade, more information on radiation leakage has become public knowledge. Despite efforts by nuclear energy institutions to downplay the threat and future consequences of the Fukushima meltdown, there has been a resurgence of popular opposition to nuclear energy. Whether or not these public attitude shift will certainly depend on the success of the Fukushima clean-up measures and nuclear safety reforms. The social and cultural tensions that have developed in Japan due to the nuclear crisis are likely to worsen in the coming years. According to previous research, the physical health effects of ionizing radiation exposure inevitably increase over time, making the long-term impact of this disaster potentially overwhelming [51,52]. They were significant barriers to public acceptance of nuclear power. However, Japanese central government considers public acceptance as the only possible solution and assumes that changes in “transparency, communication, and education” [53] (p. 1351) will ultimately result in increase in acceptance and consensus. 

In Korea, the nuclear industry has developed under a consistent interest system centered on state-owned electric utilities. An additional factor that favors the solidarity of the promoters of nuclear power is the rise of new interests, especially the export of nuclear power plants, which solidifies the policy on nuclear power. However, the Korean government reported substantial corruption in its nuclear supply chain in 2012 and 2013, with improperly certified components used in a variety of reactors, requiring unnecessary repairs. These events impaired public trust in the government’s ability to regulate nuclear energy and to reduce the errors that led to the Fukushima disaster.

Since Fukushima nuclear accidents, Korean civil activism has included a growing public awareness of nuclear safety and reluctance to consider building nuclear power plants and waste storage facilities on their doorstep. With the transition of the new administration in May 2017, and particularly with President Moon’s preference for a phase-out of nuclear energy, Korea’s nuclear energy policy today is markedly different from that of the previous administration, which was reliant on nuclear energy. The Moon administration has attempted to strengthen public support for its de-nuclear policy by establishing deliberation process with a public opinion committee on whether to postpone or resume the development of two new nuclear reactors at Sin-gori. However, the results of public opinion committee provided a divergent result: Approval for continuing development of the Sin-gori reactors while simultaneously advocating for a gradual decline in domestic nuclear power [10]. Therefore, we can recognize nuclear energy policy as a complicated, irresolvable issue.

Many previous studies used the level of acceptance of nuclear energy as a major dependent variable [8,15,53,54]. This study analyzes five different activities against the nuclear power that were measured as behaviors related to not-acceptance of nuclear energy policy: signing a petition, setting forth public appeals, making comments on government websites related to nuclear energy policy, participating in demonstrations, and donating money to support anti-nuclear activities.

### 2.4. The Role of Trust in Government and Media

The theoretical significance of this study is that it seeks the possibility and direction of usefulness of trust as main means of resolving conflicts. In particular, this study is based on psychometric paradigm in existing nuclear research, in that it focuses on socially constructed variables such as trust and affect [55,56,57,58]. Our studies focused on trust in government and media as moderator and mediator. Trust has been identified as an important factor in influencing public reactions to the location of hazardous facilities, both directly and indirectly, through risk perceptions [59,60]. In terms of risk, ‘trust’ is defined as a psychological condition in which individuals are able to accept the risk in return for positive expectations of another entity’s intentions or behavior [61]. Individuals must rely on social trust to assess risks and benefits if they have little personal knowledge of the technologies or hazards [62]. In the context of nuclear energy policy, trust in government to authorize policy formulation and implementation effectively influences the perception, especially when individuals have less experience about nuclear energy [63,64]. Therefore, the public’s perceptions of nuclear energy policy can be changed by the public’s trust, which is formed by society and their knowledge [65,66]. Moreover, previous studies have found that perceived trust has indirect effects on users’ attitudes toward and intention to employ particular technologies, via perceived benefits and risks [67], indicating that individuals’ trust could lead to a more positive perception, which could make the individuals feel lower risk and higher benefit. Trust has been investigated in relation to a wide range of technologies and products. For example, Siegrist [68] suggested significant direct effects of trust in governments or experts on public acceptance of genetic engineering or the production of genetically engineered products, as well as indirect effects on perceived risk and benefits of them. Whitfield et al. [69] indicated a significant negative effect of trust in nuclear governance on perceived risk and higher trust; lower risk perceptions with higher trust predicted positive attitudes toward nuclear power. The effect of trust on the location of high-voltage power lines are recognized in risk perception studies of natural hazards [70,71].

Trust is a multidimensional concept with having cognitive, affective, and behavioral components [68]. In addition, trust is formed through a dynamic process that occurs in a personal, institutional, and ideological context [72,73]. Other than interpersonal relationships, individuals can develop the trust in government [74] as well as in ideological values and norms [75]. Previous analysis indicates that the public’s trust in the government responsible for risk management has a substantial impact on risk communication failures [76]. Especially in areas characterized by high uncertainty, such as nuclear energy, trust plays a vital role in the success of risk communication and implementation of policies. Moreover, trust is not only a necessary precondition for successful communication, but it can also be improved by well-developed communication strategies [77]. 

This study focuses on trust in government and media. Trust in the government is critical for successfully resolving nuclear energy conflicts and influences citizens’ support for government policies toward nuclear power [78]. 

In terms of the effects of the media, numerous surveys show that media networks have considerable influence over the information they disseminate, implying that they are aggressively engaged in meaning construction [79,80]. The media have the potential to shape how the public understands and analyzes the activities of institutions by constructing the context of those actions [81]. The media are responsible for disseminating frames to a wide public and potentially amplifying their importance. When frames play an important role in social movements, in particular, the media play a role in making them work [82]. Researchers have started to examine whether media exposure results in differential cultivation effects on social and personal risk perceptions. Sussman et al. [83] found that media messages with high-risk probability and personal relevance caused participants to perceive greater personal and social risks of smokeless tobacco. As personal relevance was just one of the variables that may facilitate the media’s influence on personal risk perceptions, Tyler and Cook [84] (p. 707) urged future research to “disentangle the nature of mass media effects by probing the conditions under which mass media presentations have a personal-level impact.” Thus, previous research suggests that media may influence personal risk perceptions. In addition, according to some research, online media have a negative effect on trust in government, because they are likely to focus on negative events, such as policy conflicts or social violence, to generate attention [85], and people are inclined to pay more attention to negative aspects of news reporting [86].

Prior to the Fukushima disaster, criticism of nuclear energy in both liberal and conservative media outlets emphasized pro-nuclear subjects. Prior to the Fukushima disaster, the key pro-nuclear themes identified in the data included technical prowess, productive renewable resources, and exportable sources of energy. After Fukushima, media attention seems to have made nuclear power’s risks more apparent to the public, providing contextual messages for their analyses of high radiation risk [87].

Although there are an increasing amount of literature and surveys on trust in government and media coverage, there have been only a few comparative studies over them between countries. This study analyzes the moderated mediation or the mediated moderation role of trust in government and media in shaping relationships between belief, attitude, and behavior in nuclear energy conflicts across countries, i.e., Korea and Japan.

### 2.5. Conceptual Framework

**Figure 1:**
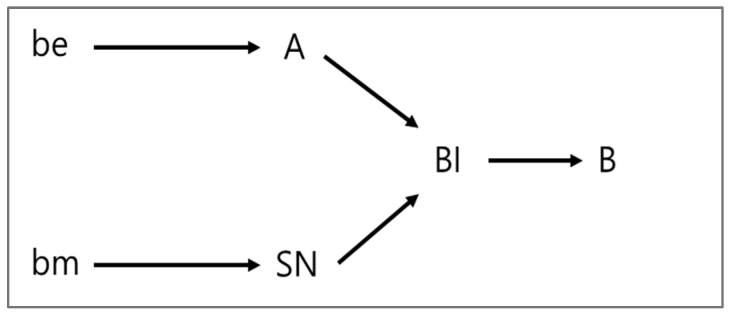
Fishbein/Ajzen model. *B = behavior*; *BI = behavioral intention*; *A = attitude*; *SN = subjective norms*; *be = beliefs about the specific consequences of behavior multiplied by the evaluation*; *bm = beliefs about the social expectations of specific others multiplied by the motivation to conform to them*.

This study approaches the traditional view of belief-attitude-behavior in terms of the opposite direction. In this study, the traditional view refers to Fishbein and Ajzen’s [34] theory on the belief–attitude–behavior relationship. Fishbein and Ajzen [34] presented a relational model between belief–attitude–intention–behavior (See Figure 1), which assumes the forward causal relationships between four components. According to them, ‘attitude’ can be described as a learned predisposition to respond in a consistently favorable or unfavorable manner with respect to a given object. In addition, ‘belief’ is a kind of information about the object. Therefore, beliefs are related to the properties of specific objects. Fishbein and Ajzen [34] explained that in the relationship between beliefs and attitudes, beliefs constitute the information base that determines attitudes. Therefore, from an information processing point of view, an individual’s attitude toward an object is based on the judge’s salient belief toward the object. Ultimately, beliefs determine attitudes; beliefs about attitudinal objects determine attitudes [34]. Finally attitudes affect behavior intentions or behaviors.

Based on previous discussion of belief, attitude, and intention and behaviors, Ajzen [13] suggests that not only beliefs and attitudes, but also social norms and sense of control can influence behavioral intentions through the TPB (Theory of Planned Behavior). Many studies [88,89] reported evidence for a forward causal relation between belief, attitude, and behavioral intention.

**Figure 2:**
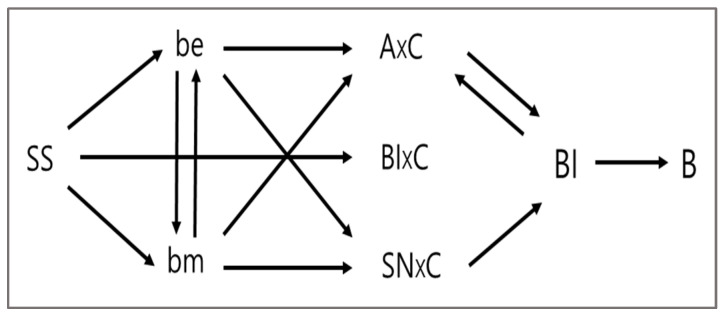
Accumulative revision of the Fishbein/Ajzen model. Source: Recited from Liska [90]. *B = behavior*; *A= attitude*; *C = contingency variables*; *BI = behavioral intention*; *SN = subjective norms*; *be = beliefs about the specific consequences of behavior multiplied by the evaluation*; *bm = beliefs about the social expectations of specific others multiplied by the motivation to conform to them*; *SS = social structure*.

However, opposite views have been presented against this traditional view. Liska [90] questioned the Fishbein/Ajzen model that specifies a recursive causal structure underlying the relationships between behavior, intentions, and attitudes. After they argued that considerable research shows that behavior affects both intentions and attitudes, they suggested an alternative model (see Figure 2) as a substitute for the traditional model. Figure 2 suggests that not only forward relationships but also interactive and reverse casual relationships can exist. In this study, we analyze reverse causal relationships as well as forward ones.

Some studies have suggested evidence about not a forward causal relationship, but a possible reverse causal relationship. For example, Fredricks and Dossett [91] demonstrated prior behavior as a direct causal influence on both subsequent behavior and behavioral intentions. Tyagi and Wotruba [92] showed that behavioral intentions may be more likely to cause one basic factor, i.e., attitude, than vice versa. Recently, Kroesen et al. [93] showed that travel attitudes and behaviors mutually influence each other over time. Moreover, contrary to Fishbein/Ajzen’s assumptions, behavior influences attitude more than vice versa. Sussman and Gifford [94] demonstrated that one’s formed behavioral intentions may affect the attitude and norm in a reverse-causal direction.

A key topic in this study is the issue of causality between variables. The causality is relative subjective concepts which can be varied according to different points of view. King et al. [95] defined ‘causality’ as a case in which a change in the value of one variable is related to a change in the value of another variable in a specific environment (a case in which such a change occurs without a change in the other variable). John Sturt Mill [96] argued that the traditional principle of causality is that (1) cause precedes effect in time, (2) cause and effect are related, and (3) alternative explanations are removed by explaining cause–effect. This last definition excludes alternative hypotheses about actual causes and effects. On the other hand, recently, King et al. [95] pointed out three conditions for causality. First, correlation or covariance exist between X and Y; second, there is no reverse relationship from Y → X; and third, the change is not induced by other variables. All of these definitions assume a strong positivist claim of which the cause should be observed. In this vein, Russell [97] asserts that existing scientists criticize the unobserved as a cause. 

About discussion over causality, we agreed partly with the positivists in terms of the importance of empirical observation of causal rules which they strongly presented. However, we agreed with Cook and Campbell’s [98] criticism of the strong positivists, pointing out that “they deny the number of different cases of causation just because they have not been observed.” Additionally, we do not agree with the denial of reverse causality as a condition of causality. In other words, we consider that the possibility of reverse causal relationship should be acknowledged until the certain causal relationship is confirmed. In this respect, this study examined the possibility of reverse causal relationship. In the traditional model, beliefs influence attitudes and attitudes influence behavior. However, in this study, in the low involvement model, attitude affects beliefs, and in the hedonic model, behavior affects beliefs and attitude. Further, we agree with the Essentialist Theories’ argument (see [98]) that the cause does not necessarily need temporal pre-existence of cause and that it can be simultaneous. This is because it is difficult to recognize temporal pre-existence until a definitive causal relationship is observable. Such open assumptions about causality make it possible to test new causal relationships, i.e., reverse causal relationships.

**Figure 3:**
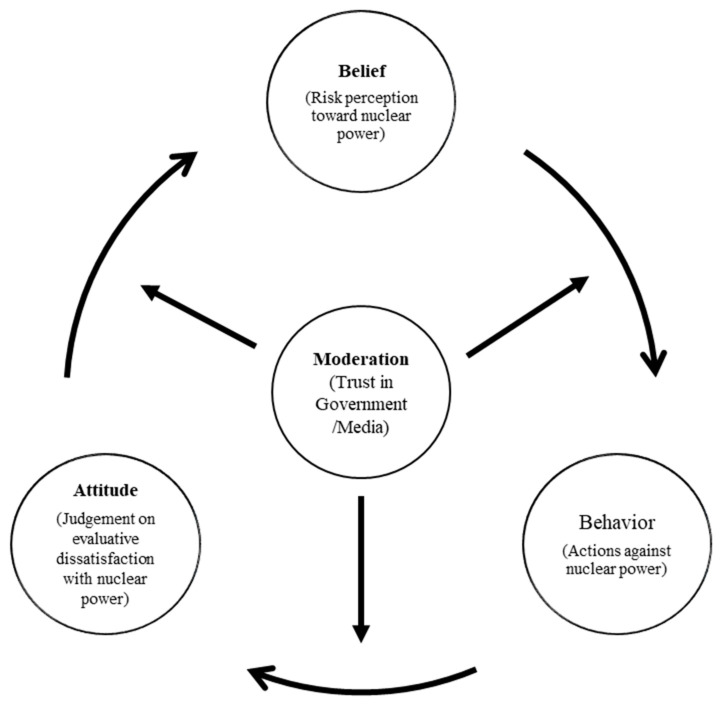
Conceptual framework of the relationship between belief, attitude, and behavior moderated by trust in government and media.

This study focuses on the causal pathway from belief (perception), attitude (judgment), and behavior (actions against nuclear power, i.e., not-acceptance). Also, this study focuses on the mediated moderation and moderated mediation effects. Two effects hold the opposite attributes. Mediated moderation refers to the process in which the moderating effect is transmitted through a mediator [99]. Given that there is an overall moderating effect on the relationship between a predictor and an outcome variable, mediated moderation is concerned with how the moderator exerts its effect on the relationship between the predictor and outcome. Figure 3 summarizes the research framework for this study, which is based on three models (high, low, and hedonic models). It essentially consists of three components: belief (risk perception toward nuclear power), attitude (judgment of evaluative dissatisfaction with nuclear power), and behavior (actions against nuclear power). The control variables included gender, age, level of education, and income.

**Figure 4:**
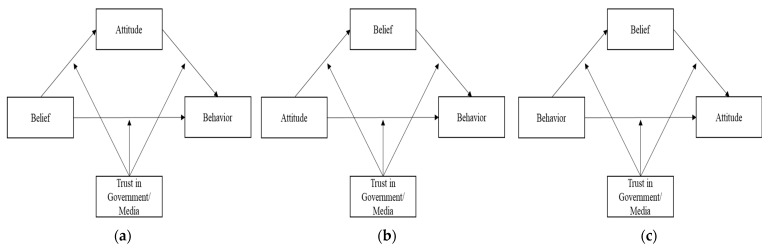
The three models (high involvement (**a**), low involvement (**b**), and hedonic model (**c**)) with trust in government and media.

To verify the above conceptual framework, three different models in Figure 4 were tested according to different contexts, that is, Korea and Japan. Unlike the traditional model, which assumes linear relationships among belief–attitude–behavior, this study proposes a more interactive and dynamic model to analyze how trust acts as a moderated mediator and mediated moderator.

**Figure 5:**
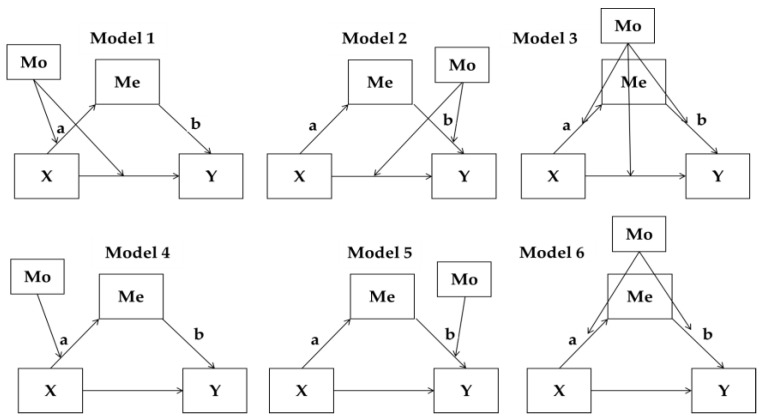
Six different patterns of three models with mediated moderation (Models 1~3) and moderated mediation (Models 4~6).

Based on these three models, Muller et al. [22] developed a model to determine the relationship between independent variables, mediation variables, moderation variables, and dependent variables. Furthermore, based on this integrated model, we identified the following six patterns in Figure 5. The first three patterns are mediated moderation models. The other three patterns are moderated mediation models. As mentioned before, this study focuses on the moderated mediation or mediated moderation effects of trust in government and media on the relationships between belief, attitude, and behavior. Therefore, either mediated moderation or moderated mediation would be detected, and the six different patterns of models could be deployed as follows in Figure 5.

The basic equation model for verifying the six models is as follows.

(1) $$Y=\beta_{10}+\beta_{11}X+\beta_{12}Mo+\beta_{13}XMo+\varepsilon_{1}$$

(2) $$Me=\beta_{20}+\beta_{21}X+\beta_{22}Mo+\beta_{23}XMo+\varepsilon_{2}$$

(3) $$Y=\beta_{30}+\beta_{31}X+\beta_{32}Mo+\beta_{33}XMo+\beta_{34}Me+\beta_{35}MeMo+\varepsilon_{31}$$

## 3. Sample and Measurement

### 3.1. Sample

This study was based on data from national online surveys of citizens living in Korea and Japan. The data was collected in 2018 (May and June). The online survey (structured questionnaire) known as the “Nuclear Power Policy Survey for the Public” measured perceived risk, attitude toward nuclear energy policy, level of acceptance of nuclear power, and other nuclear energy policy-related factors. Data were collected from adult men and women aged 19–59 years. 500 people were surveyed through an online panel survey in Korea, and 527 people were surveyed in Japan as well.

### 3.2. Measurement

In this study, three main variables are defined as Belief—Perceived Risk, the extent to which citizens report their perception on the level of risk of nuclear energy; Attitude—Judgment, the extent to which citizens dissatisfied with nuclear policy; and Behavior—Not-Acceptance, the extent to which citizen reported they acted against the nuclear power in each area. The three dependent variables were as follows:

Belief—Perceived Risk: This concept was measured using the following six questions (1) how much one agrees with the level of risk of using nuclear plants for providing electricity; (2) the level of accidents in nuclear facilities; (3) the overall level of danger of nuclear energy; (4) the level of risk in nuclear energy in terms of economic issues; (5) the level of nuclear risk regarding environmental problems; (6) the level of risk related to usability such as medical, food, etc. (scale: 1 = strongly disagree, 2 = disagree, 3 = neutral, 4 = agree, and 5 = strongly agree). These questions were combined into an additive construct (Cronbach’s α = 0.832 in Korea and 0.847 in Japan).

Attitude—Judgment of Nuclear Policy: This notion was measured using the following six questions: (1) Currently, what is the extent to which one agrees with the level of affection toward the overall system and policies? (2) How much does one agree with the extent of nuclear energy as a major energy source? (3) What is the current level of attitude toward (trust in) the current nuclear policy and institutes? (4) What is the current level of dissatisfaction with nuclear policy in terms of safety? (5) What is the current level of dissatisfaction with nuclear policy in terms of usability? (6) What is the current level of dissatisfaction with nuclear policy in terms of environmental friendliness (scale: 1 = strongly disagree, 2 = disagree, 3 = neutral, 4 = agree, and 5 = strongly agree). These questions were combined into an additive construct (Cronbach’s α = 0.844 in Korea and 0.891 in Japan).

Behavior—Not-Acceptance of Nuclear Energy: This notion was measured by the following five questions: (1) what is the level of agreement that one would like to sign the petition on anti-nuclear requests to the government; (2) to set forth public appeals; (3) to make comments on government or related websites; (4) to participate in nuclear meetings and protests; and (5) to donate money to support politicians who oppose nuclear energy-friendly policies (scale: 1 = strongly disagree, 2 = disagree, 3 = neutral, 4 = agree, 5 = strongly agree). These questions were combined into an additive construct (Cronbach’s α = 0.873 in Korea and 0.912 in Japan).

Trust in the government and trust in the media were measured using a five-point scale. Trust in the government was measured through eleven items, and trust in the media was measured through four items.

Table 1 contains further details of the measurers. All respondents were well represented by variables that were subjected to reliability analysis (see Table 1). The survey questions were selected so that the constructs were as similar as possible to those in prior studies [11,24,25].

Table 2 shows the differences in belief (risk perception), attitude (judgment on evaluative satisfaction with nuclear power), behavior (actions against nuclear power), and trust in government and media between Korea and Japan. Among the six items of belief (risk perception), three items were higher in Korea, and three items were higher in Japan. In attitude (judgment on evaluative dissatisfaction with nuclear power), Japan was higher than Korea on five measurement items and lower on one item. However, in the case of behavior (actions against nuclear power), Korea was higher than Japan. These results show that satisfaction and opposition behaviors differ. Trust in government and media is higher in Korea than in Japan. The risk perceptions in Korea and Japan are similar, but there are differences between the two countries in terms of dissatisfaction, lack of acceptance, and trust. Compared to Korea, Japan has a higher level of dissatisfaction with nuclear power policies, a lower level of opposition action and trust in the government. It is interesting to note that, in Japan, a high level of dissatisfaction and a low level of opposition action lead to low trust in the government. From these results, it can be inferred that the level of trust in the government is high when there is a lower level of dissatisfaction and higher opposition to the nuclear issue. Trust in the government seems to be high because it is highlighted as a problem solver when there is high resistance behavior.

## 4. Analysis

### 4.1. Discriptive Analysis

Table 3 shows the differences between Korea and Japan in terms of belief, attitude, and behavior according to demographic variables. When viewing only statistically significant cases, women in Korea have a higher risk perception than men. By age, in Korea, young people have a high risk perception, but in Japan, older people have a higher risk perception than younger people. In terms of attitude, men in Korea show higher dissatisfaction than women. In terms of age, in Japan, older people are more dissatisfied than younger people. Finally, regarding behavior, in both Korea and Japan, older people have higher oppositional behavior than young people. When looking at the results of the above analysis comprehensively, in Korea, compared to women, men have a high level of both risk perception and dissatisfaction with nuclear energy, and their anti-nuclear behavior is also high. In Japan, older people have higher risk perception and dissatisfaction than younger people, and at the same time have higher anti-nuclear activity. In this case, a high level of risk perception and dissatisfaction increases opposition to nuclear power.

To see the dependence of behavior and attitude on the level of trust in mass media, we conducted an ANOVA test. The results are listed in Table 4. In Japan, when trust in government is high, risk perception is low, but the opposite result is shown in Korea: when trust in the government is high, risk perception is also high. In Japan, dissatisfaction is high among groups with low trust in the government. In anti-nuclear-related actions, high trust in the government in Japan is linked to low opposition actions, but in Korea, it increases opposition actions. In both Korea and Japan, even when trust in the media is high, opposition toward the nuclear energy is high. From the above results, it can be seen that there is a difference in the effect of trust in the government between Korea and Japan. Additionally, contrary to common sense, there are some cases in which high trust can induce high-risk perception and opposition behavior.

### 4.2. Effect of Trust in Government

This study employed Muller et al. [22]’s model to determine how citizens in both countries react to nuclear energy policy. Using the verifying model, the series of regression statistical outputs tell us which pattern fits the test model. All three models were tested.

**Figure 6:**
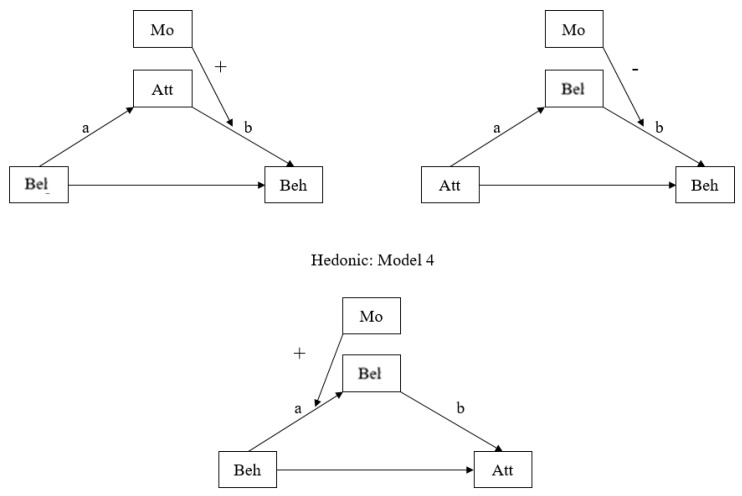
Government Trust Model in Korea.

**Figure 7:**
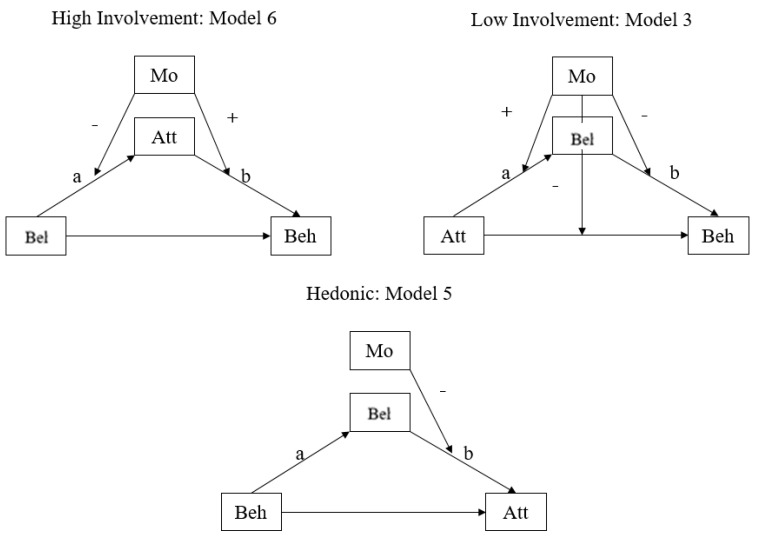
Government Trust Model in Japan.

The results are displayed for different values of the dependent variable in Table 5 and Table 6, as well as Figure 6 and Figure 7. Statistically significant coefficients in Korea, are showed in Table 5. These valid coefficients represent the influences that were moderated. All three models met one of six patterns. Thus, as shown in Figure 6, citizens in Korea used three patterns of decision-making regarding nuclear energy issues, but the impact of trust in government was somehow different in those models.

In particular, all three patterns were moderated mediation models. For high-and low-involvement cases, Model 5 was found, whereas in the hedonic case, Model 4 was found. This means that the level of trust in the government in Korea is more likely to influence Korean citizens’ decisions about their actions, in other words, their behavior.

Figure 6 shows that in Korea, not only general causal relationships (belief → attitude → behavior) but also reverse causal relationships (attitude → belief → behavior or behavior belief → attitude) can exist. In addition, it can be seen that the role of trust in the government varies according to the three suggested models. For example, in the high-involvement model, trust in the government moderates the relationship between attitude and behavior, whereas in the low-involvement model, trust in the government moderates the relationship between belief and behavior.

Meanwhile, as we can see in Table 6 and Figure 7, citizens in Japan showed different outcomes. Three patterns were utilized in the same way as in Korea. However, the detailed results are different. First, there was a different mediation and moderation effect in the case of low involvement across two countries. Per high-involvement and hedonic cases, people in two countries used moderated mediation models (Models 5 and 6). However, in low involvement, Koreans used the moderated mediation model whereas Japanese used a mediated moderation model (Model 3). Additionally, the moderation role of government in trust varies across two countries. For example, in the case of the hedonic model, trust in the government intervenes the relationships between behavior and beliefs in Korea whereas it intervenes the relationship between belief and behavior in Japan. 

The level of trust in the government influences citizens’ behavior at the same time as their attitude and belief. This means that Japanese citizens are more likely to consider trust in government as a factor not only in their decisions on action but also for their belief and attitude. The effect of trust in the government on moderating citizens’ decision-making processes was modeled in both Korea and Japan. However, the detailed patterns differed. Japan has experienced a real accident at a nuclear energy plant. This may be the cause of these differences. Korean citizens’ beliefs and attitudes are less likely to be influenced by their level of trust in the government, while trust in the government has a greater effect on Japanese citizens’ belief and attitude. These differences can provide insights into the government leadership of both countries. Without understanding the effects of public trust in government, governments cannot enhance citizens’ level of satisfaction with nuclear energy policies.

### 4.3. Effect of Trust in Media

**Figure 8:**
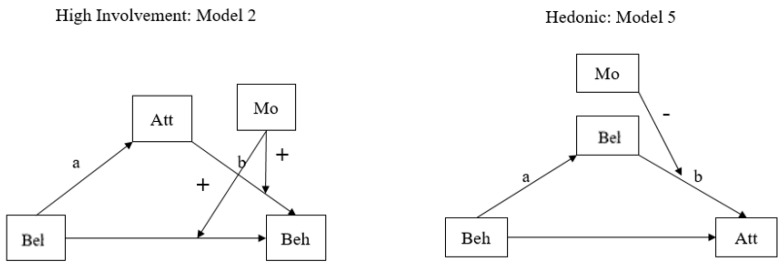
Media Trust Model in Korea.

**Figure 9:**
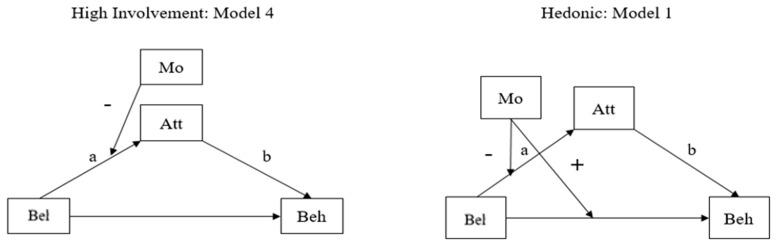
Media Trust Model in Japan.

In the case of the influence of trust in media, two patterns were statistically significant in Korea. The high-involvement and hedonic models were significant. Table 7, Figure 8 in Korea and Table 8, Figure 9 in Japan show that not only general logical causal relationships (belief → attitude → behavior) but also reverse causal relationships (attitude → belief → behavior or (behavior belief → attitude) can exist across two countries.

Viewing from moderation and mediation effect, in the case of high involvement, a mediated moderation model (Model 2) was detected. In the hedonic case, a moderated mediation model (Model 5) was found in Korea. In Japan, similar to Korea, two cases of high-involvement and hedonic cases were detected. However, the details are somewhat different. Per high involvement, Japanese citizens used a moderated mediation model (Model 4), and, in the hedonic case, they followed a mediated moderation model (Model 1).

By using the same verifying model as the one used for the effect of trust in the government, the regression results show us which patterns fit into the models. Both Korean and Japanese citizens used only two models: high-involvement and hedonic models. In general, trust in the media has an effect on citizens’ beliefs and attitudes.

However, interestingly, the detailed sub-patterns are the opposite in Korea and Japan. In the high involvement model, Korean citizens took influence from the media on the relationship between belief and behavior, as well as attitude and behavior. Yet, Japanese citizens had an impact from the media on the relationship between belief and attitude. Meanwhile, in the hedonic model, Japanese citizens had the relationship between belief and behavior, belief+ and attitude. Korean citizens had an impact from media on the relationship between belief and attitude.

Thus, the general patterns could be found in both countries, but the details often oppose each other. One possible explanation could be the differences in the strategies of delivering messages between Korea and Japan as well as the level of general public trust. According to the 2020 Edelman Trust Barometer, Korea scored 50 out of 100, which is at the neutral level (50–59), and Japan scored 42 out of 100, which is at the distrust level (1–49) [100]. In addition, in 2021, according to the World Press Freedom Index, Korea ranked 42th, and Japan ranked 67th [100]. This cannot explain all the reasons for the differences between the two countries, but it might provide a clue to interpret the empirical findings.

## 5. Summary

We questioned simple forward causal relationships between belief, attitude, and behavior and tested the reverse causal relationships between them and the role of intervening variable, i.e., trust in this study, in those relationships. This study, in detail, includes three research questions: (1) Is the causal relationship between beliefs–attitude–behavior empirically working as described from a traditional point of view? (2) How does trust in government and media as mediators or moderators affect the causal relationships between belief, attitude, and behavior? (3) Is the causal structure of trust influenced differently across countries, specifically Korea and Japan?

Our findings indicate that first, there are not only general forward relationships but also reverse relationships between belief, attitude, and behavior. Second, there exist the moderated mediation and mediating moderation effects of trust in the government and media across two countries. Third, the effects of trust in the government and media work significantly overall in both countries. Such interactions differ across two countries.

## 6. Conclusions

This study highlights the use of ‘bounded rational’ approaches in citizens’ decision-making processes regarding nuclear energy policy. As it is difficult to institutionalize completely neutral design with full rationality, we adapted the notion of bounded rationality that admits limited rational choices [101,102]. As there are many circumspect factors that limit the complete rational choices of individuals, citizens normally adopt heuristics to make difficult decisions or select high-stakes choices through low-powered incentives [103]. In this regard, we used the notion of bounded rationality as the basis of our analysis to investigate the reverse causal relationships and mediated moderation/moderated medication effects.

The results of this study can be summarized as follows. First, it was found that there can exist a model that assumes not only forward causality in the high-involvement model (belief → attitude → behavior) which assumes a traditional rational model, but also reverse causal relationships in the low-involvement model (attitude → belief → behavior) or hedonic mode (behavior → belief → attitude). Second, the moderating or mediating role of trust may vary depending on the contexts. Even in the same model in Korea and Japan, it can be seen that trust intervenes in different positions in the relationships between variables and takes different roles, i.e., positive or negative effect. For example, in the same high-involvement model, trust in media produced a moderated mediation effect in Korea, but a mediated moderation effect in Japan. Third, two types of trust work differently according to the suggested models. In the case of trust in the government, it works in three models, whereas in the case of media trust, it works in two models: the high-involvement model and the hedonic model.

Prior studies have examined the connection between three components, i.e., belief, attitude, and behavior, only in terms of a rational approach and general forward causal relationships, but they have not explored the reverse relationships between them by using different investigational methods. We confirmed the reverse relationships between three variables. Overall, like Mok and his colleagues’ study [104], our study showed individual citizens used different psychological models and strategies to judge the risk of nuclear energy polices. Also, by using three different models, we examined the effects of trust in the government and the media as mediated moderation or moderated medication factors in nuclear energy usage in the two countries. Through these comparisons, we found that the level of trust in government affects all three different models of citizens’ decision-making processes in both Korea and Japan. However, the detailed patterns differed. Korean citizens’ belief and attitudes are less likely to be influenced by the level of trust in the government. Japanese citizens experience a greater effect of trust in government on their belief and attitudes. This might explain the effects of different cultures and experiences on nuclear energy policies. 

This study implies that to manage the acceptance of nuclear power, it considers the causality, mediation/moderation, and context. That work is not easy, but that needs. 

A limitation of this study is that when making model, we dismissed the role of significant variables. First, one needs to consider the contextual or structural variables in constructing the model. Although Korea and Japan are close countries, they have different cultures and values in many respects. If culture and value variables can be included in the model, it helps one to find the influence of endogenous factors embedded in the two countries. Second, in terms of variables, this study mainly focused on four variables: perceived risk, policy satisfaction, policy support, and trust. In the psychometric paradigm, emotions and knowledge are considered important variables affecting risk-related judgments, but this study did not reflect them in the model. It is necessary to construct a model that considers the impact of emotion and knowledge on policy satisfaction and policy support. Third, trust in the current model consists of two dimensions, i.e., trust in the government and media. It is necessary to measure trust in more diverse actors and then analyze the influence of such multidimensionality on trust. Fourth, nuclear-related issues should be considered because they are greatly affected by disaster experiences. The Fukushima nuclear accident brought about a fundamental change in thinking about nuclear power. From an empirical point of view, the impact of disaster experience on policy satisfaction and support should be analyzed. Moreover, other factors such as political preference and social solidarity could influence the relationships in the investigative models in this study. Overall, these future studies will help us better understand how citizens communicate and act when they are at risk.

## Figures and Tables

**Table 1:** Frequency of respondents.

Variables	Category	Korea *N* (%)	Japan *N* (%)
Gender	Male	256 (51.2)	285 (49.8)
	Female	244 (48.8)	287 (50.2)
Age	19–29	116 (23.2)	110 (19.2)
	30–39	114 (22.8)	142 (24.8)
	40–49	136 (27.2)	165 (28.8)
	Over 50	134 (26.8)	155 (271)
Education	Doctorate	6 (1.2)	7 (1.2)
	Master’s	43 (8.6)	20 (3.5)
	Bachelor’s	342 (68.4)	313 (54.7)
	Junior College	70 (14)	26 (4.5)
	High School & Below	158 (15.2)	206 (36.0)
Household Income	Less than 2 million (M) won	42 (8.4)	78 (13.6)
	2 M won	62 (18.6)	107 (18.7)
	3 M won	93 (18.6)	97 (17.0)
	4 M won	89 (17.8)	95 (16.6)
	5 M won	82 (17.8)	65 (11.4)
	6 M won	52 (10.4)	46 (8.0)
	7 M won	26 (5.2)	34 (5.9)
	8 M won & above	52 (10.8)	50 (8.7)

**Table 2:** Measures of Variables.

*Composite Name*	*Alpha Values*	*Survey Questions*	*Korea* *(Mean/SD)*	*Japan* *(Mean/SD)*
Belief *(Risk perception toward nuclear power)*	0.832 ^a^/0.847 ^b^	I think that I admit the high risk of nuclear power as I rely on nuclear energy.	3.46/0.95	3.56/1.05
		2. I think that it is risky that nuclear plants are constructed near my residential area.	3.75/0.95	3.89/1.00
		3. Basically, nuclear power is dangerous.	3.70/0.97	3.81/1.00
		4. I think there is potential risk of nuclear power in terms of economy.	3.60/0.87	3.36/0.98
		5. I think there is potential risk of nuclear power in terms of environment.	3.79/0.90	3.33/0.93
		6. I think there is potential risk of nuclear power in terms of usability.	3.57/0.88	3.16/0.84
Attitude *(Judgment on evaluative dissatisfaction with nuclear power)*	0.844 ^a^/0.891 ^b^	Basically, I have a negative attitude toward nuclear power.	3.14/0.96	3.23/1.07
		2. I oppose the use of nuclear power as a major energy source.	3.23/0.85	3.25/1.09
		3. I distrust the overall nuclear policies and institutes.	3.42/0.85	3.39/0.97
		4. I am dissatisfied with public policies related to nuclear safety.	3.22/0.84	3.54/0.93
		5. I am dissatisfied with public policies related to nuclear usability.	3.21/1.0	3.35/0.90
		6. I am dissatisfied with public policies related to nuclear eco-friendliness.	3.26/0.99	3.22/0.82
Behavior *(Actions against nuclear power)*	0.873 ^a^/0.912 ^b^	I sign the petitions that are opposed to nuclear energy.	2.77/0.99	2.74/1.12
		2. I make appeals that are opposed to nuclear energy.	2.75/0.97	2.69/1.00
		3. I post comments on governmental or related website to oppose nuclear energy.	2.66/1.0	2.52/0.97
		4. I participate in anti-nuclear meetings or protests.	2.61/0.99	2.56/1.08
		5. I donate money to politicians who are opposed to nuclear energy.	2.40/0.99	2.53/1.08
Trust in government	0.919 ^a^/0.981 ^b^	I think that the government understands nuclear conflicts clearly and accurately.	2.94/0.91	2.92/0.90
		2. I think that the government is fair and neutral on nuclear conflicts.	2.91/0.86	2.67/0.96
		3. I think that governmental nuclear polices are established based on public interest.	2.76/0.92	2.85/0.93
		4. I think that the government has expertise on coordination and negotiation to solve nuclear conflicts.	2.93/0.93	2.57/0.86
		5. I think that the government has power to resolve nuclear conflicts.	2.98/0.93	2.52/0.89
		6. I think that the government has power to resolve nuclear conflicts.	2.96/0.93	2.48/0.86
		7. I think that the government has a willingness to share information related to nuclear power energy development.	2.89/0.87	2.5/0.89
		8. I think that the government has a willingness to listen to stakeholders in nuclear conflicts.	2.99/0.84	2.74/0.94
		9. I think that the government has interacted with stakeholders to resolve nuclear conflicts.	2.89/0.85	2.73/0.90
		10. I think that the government improves nuclear policies after listening to citizens’ voices.	2.98/0.80	2.51/0.87
		11. I think that the government implements agreements well which are outcomes of consensus on nuclear issues.	2.88/0.90	2.59/0.87
Trust in media	0.771 ^a^/0.768 ^b^	I trust the information from TV and radio media.	3.0/0.85	2.80/0.96
		2. I trust in information from traditional media (newspapers and magazines).	2.9/0.85	2.87/0.94
		3. I trust in information from my family, friends, and neighbors.	3.26/0.90	3.04/0.80
		4. I trust in information from emerging information technologies (Internet, YouTube, SNS, etc.)	2.92/0.90	2.78/0.86

a = Korea, b = Japan.

**Table 3:** ANOVA Test on Belief, Attitude, and Behavior by Gender, Age, Education, and Income.

			Korea		Japan	
			Means/SD	F (df)	Means/SD	F (df)
Belief *(Risk perception toward nuclear power)*	Gender	Women	3.52/0.75	17.295 (499) ***	3.63/0.72	0.439 (571)
		Men	3.77/0.55		3.40/0.73	
	Age	Older	3.58/0.70	4.136 (499) *	3.59/0.74	6.534 (571) **
		Younger	3.70/0.64		3.43/0.71	
	Education	Higher	3.67/0.68	2.785 (499)	3.53/0.76	0.387 (571)
		Lower	3.54/0.66		3.49/0.68	
	Income	Higher	3.62/0.68	0.655 (499)	3.57/0.74	3.272 (571)
		Lower	3.67/0.66		3.46/0.73	
Attitude *(Judgment on evaluative dissatisfaction with nuclear power)*	Gender	Women	3.11/0.70	22.143 (499) ***	3.40/0.75	5.510 (571) **
		Men	3.39/0.62		3.25/0.79	
	Age	Older	3.21/0.66	1.324 (499)	3.39/0.81	4.389 (571) *
		Younger	3.28/0.69		3.26/0.73	
	Education	Higher	3.27/0.67	3.168 (499)	3.35/0.79	0.678 (571)
		Lower	2.14/0.70		3.29/0.79	
	Income	Higher	3.24/0.65	0.004 (499)	3.30/0.73	0.693 (571)
		Lower	3.24/0.71		3.35/0.82	
Behavior *(Actions against nuclear power)*	Gender	Women	2.56/0.86	4.919 (499) *	2.63/0.83	0.430 (571)
		Men	2.72/0.74		2.58/0.93	
	Age	Older	2.63/0.76	0.000 (499)	2.71/0.91	6.364 (571) **
		Younger	2.63/0.85		2.51/0.86	
	Education	Higher	2.67/0.82	3.446 (499)	2.62/0.93	0.406 (571)
		Lower	2.51/0.76		2.58/0.82	
	Income	Higher	2.62/0.84	0.191 (499)	2.61/0.93	0.008 (571)
		Lower	2.65/0.75		2.50/0.84	

* *p* < 0.05, ** *p* < 0.01, *** *p* < 0.001.

**Table 4:** ANOVA Test on Belief, Attitude, and Behavior.

			Korea		Japan	
			Means/SD	F (df)	Means/SD	F (df)
Cogntion *(Risk perception toward nuclear power)*	Trust in G	Low group	3.50/0.72	18.482 (499) ***	3.82/0.73	91.069 (571) ***
		High Group	3.76/0.60		3.27/0.63	
	Trust in M	Low group	3.64/0.73	0.024 (499)	3.47/0.83	2.053 (571)
		High Group	3.64/0.62		3.55/0.64	
Attitute *(Judgment on evaluative dissatisfaction with nuclear power)*	Trust in G	Low group	3.23/0.71	0.259 (499)	3.64/0.84	84.712 (571) ***
		High Group	3.26/0.64		3.07/0.61	
	Trust in M	Low group	3.22/0.72	0.536 (499)	3.27/0.90	2.690 (571)
		High Group	3.26/0.63		3.37/0.65	
Behavior *(Actions against nuclear power)*	Trust in G	Low group	2.42/0.75	33.823 (499) ***	2.69/0.96	4.537 (571) *
		High Group	2.83/0.80		2.53/0.82	
	Trust in M	Low group	2.49/0.77	13.941 (499) ***	2.42/0.92	20.097 (571) ***
		High Group	2.76/0.82		2.75/0.89	

* *p* < 0.05, *** *p* < 0.001.

**Table 5:** Government Trust Model in Korea.

	High Involvement Model: Moderated Mediation				Low Involvement Model: Moderated Mediation				Hedonic Model: Moderated Mediation		
**Model 5**	DV: Behavior b (t)	DV: Attitude b (t)	DV: Behavior b (t)	**Model 5**	DV: Behavior b (t)	DV: Belief b (t)	DV: Behavior b (t)	**Model 4**	DV: Attitude b (t)	DV: Belief b (t)	DV: Attitude b (t)
X: Attitude	0.503 (3.005) **	0.604 (4.675) **	0.618 (3.234) **	X: Attitude	0.254 (1.724)	0.761 (6.184) **	−0.138 (−0.726)	X: Behavior	0.543 (4.625) **	0.660 (5.255) **	0.181 (1.368)
Mo: Gov Trust	0.508 (2.369) *	−0.097 (−0.588)	0.341 (1.741)	Mo: Gov Trust	0.009 (0.053)	0.454 (3.310) **	0.341 (1.741)	Mo: Gov Trust	−0.087 (−0.866)	0.466 (4.323) **	−0.257 (−1.733)
XMo: X * Mo	−0.044 (−0.746)	0.005 (0.108)	−0.213 (−3.232) **	XMo: X * Mo	0.124 (2.791)	−0.068 (−1.637)	0.255 (4.009) **	XMo: X * Mo	−0.016 (−0.438)	−0.125 (−3.097) **	0.057 (1.355)
Me: Attitude			−0.138 (1.726)	Me: Belief			0.618 (3.234) **	Me: Belief			0.558 (3.678) **
MeMo: Me * Mo			0.255 (4.009) **	MeMo: Me * Mo			−0.213 (−3.232) **	MeMo: Me * Mo			−0.025 (−0.476)

* *p* < 0.05, ** *p* < 0.01.

**Table 6:** Government Trust Model in Japan.

	High Involvement Model: Moderated Mediation				Low Involvement Model: Mediated Moderation				Hedonic Model: Moderated Mediation		
**Model 6**	DV: Behavior b (t)	DV: Attitude b (t)	DV: Behavior b (t)	**Model 3**	DV: Behavior b (t)	DV: Belief b (t)	DV: Behavior b (t)	**Model 5**	DV: Attitude b (t)	DV: Belief b (t)	DV: Attitude b (t)
X: Attitude	0.528 (12.276) **	0.793 (30.454) **	−0.185 (−0.939)	X: Belief	0.544 (3.722) **	1.083 (12.090) **	−0.162 (−0.702)	X: Behavior	0.944 (8.976) **	0.693 (6.667) **	0.383 (4.243) **
Mo: Gov Trust	0.197 (4.229) **	0.095 (3.346) **	0.171 (3.675) **	Mo: Gov Trust	0.201 (1.063)	0.292 (2.512) *	0.070 (0.384)	Mo: Gov Trust	0.357 (3.623) **	0.260 (2.674) **	0.255 (2.309)
XMo: X * Mo	0.060 (4.220) **	−0.030 (−3.498) **	0.282 (3.775) **	XMo: X * Mo	0.014 (0.264)	−0.086 (−2.675) **	0.119 (1.509)	XMo: X * Mo	−0.159 (−4.499)	−0.098 (−2.814) **	−0.069 (−2.294)
Me: Belief			0.766 (3.848) **	Me: Attitude			0.681 (3.474) **	Me: Belief			0.841 (7.926) **
MeMo: Me * Mo			−0.214 (−2.937)**	MeMo: Me * Mo			−0.070 (−1.040)	MeMo: Me * Mo			−0.039 (−1.033)

* *p* < 0.05, ** *p* < 0.01.

**Table 7:** Media Trust Model in Korea.

	High Involvement Model: Mediated Moderation				Hedonic Model: Moderated Mediation		
**Model 2**	DV: Behavior b (t)	DV: Attitude b (t)	DV: Behavior b (t)	**Model 5**	DV: Attitude b (t)	DV: Belief b (t)	DV: Attitude b (t)
X: Attitude	−0.133 (−0.620)	0.654 (3.972) **	−0.129 (−0.528)	X: Behavior	0.296 (2.234) *	0.322 (2.230) *	0.022 (0.177)
Mo: Media	−0.555 (−1.962) *	0.081 (0.372)	−0.717 (−2.767) **	Mo: Media	−0.292 (−2.456) *	−0.143 (−1.103)	0.222 (1.127)
XMo: X * Mo	0.226 (2.962) **	−0.021 (−0.368)	0.106 (1.270)	XMo: X * Mo	0.060 (1.40B7)	0.012 (0.251)	0.102 (2.493) *
Me: Attitude			0.042 (0.197)	Me: Belief			0.868 (5.314) **
MeMo: Me * Mo			0.176 (2.499) *	MeMo: Me * Mo			−0.155 (−2.698) *

* *p* < 0.05, ** *p* < 0.01.

**Table 8:** Media Trust Model in Japan.

	High Involvement Model: Moderated Median				Hedonic Model: Mediated Moderation		
**Model 4**	DV: Behavior b (t)	DV: Attitude b (t)	DV: Behavior b (t)	**Model 1**	DV: Behavior b (t)	DV: Attitude b (t)	DV: Behavior b (t)
X: Behavior	0.376 (4.437) **	0.365 (4.423) **	−0.220 (−2.991) **	X: Belief	0.554 (4.266) **	1.165 (14.972) **	0.699 (3.001) **
Mo: Media	−0.454 (−5.037) **	−0.397 (−4.521) **	0.398 (4.116) **	Mo: Media	0.044 (0.253)	0.395 (3.757) **	−0.183 (−1.105)
XMo: X * Mo	0.040 (1.268)	0.018 (0.580)	0.165 (5.907) **	XMo: * Mo	0.050 (1.022)	−0.132 (−4.538) **	−0.179 (−2.104) *
Me: Belief			1.371 (15.233) **	Me: Attitude			−0.246 (−1.242)
MeMo: Me * Mo			−0.269 (−7.822) **	MeMo: Me * Mo			0.315 (4.188) **

* *p* < 0.05, ** *p* < 0.01.

## Data Availability

The data presented in this study are available upon request from the corresponding author.
